# Identification of Anti-Persister Activity against Uropathogenic *Escherichia coli* from a Clinical Drug Library

**DOI:** 10.3390/antibiotics4020179

**Published:** 2015-05-12

**Authors:** Hongxia Niu, Peng Cui, Wanliang Shi, Shuo Zhang, Jie Feng, Yong Wang, David Sullivan, Wenhong Zhang, Bingdong Zhu, Ying Zhang

**Affiliations:** 1 Department of Molecular Microbiology and Immunology, Bloomberg School of Public Health, Johns Hopkins University, Baltimore, MD 21205, USA; E-Mails: niuhongxia1985@163.com (H.N.); keanuc@163.com (P.C.); wshi3@jhu.edu (W.S.); shuozhang66@gmail.com (S.Z.); jfeng16@jhu.edu (J.F.); dsulliv7@jhmi.edu (D.S.); 2 Lanzhou Center for Tuberculosis Research and Institute of Pathogenic Biology, School of Basic Medical Sciences, Lanzhou University, Lanzhou 730000, China; E-Mail: bdzhu@lzu.edu.cn; 3 Key Laboratory of Medical Molecular Virology, Department of Infectious Diseases, Huashan Hospital, Shanghai Medical College, Fudan University, Shanghai 200040, China; E-Mail: zhangwenhong@fudan.edu.cn; 4 Department of Clinical Microbiology Lab, Provincial Hospital Affiliated to Shandong University, Jinan 250021, China; E-Mail: sdwangyong@126.com

**Keywords:** *Escherichia coli*, persisters, anti-persister activity, clinical drug library, urinary tract infection

## Abstract

Uropathogenic *E. coli* is a major cause of urinary tract infections (UTIs), but current antibiotics do not always effectively clear the persistent infection. To identify drugs that eliminate uropathogenic *E. coli* persisters, we screened a clinical drug library consisting of 1524 compounds using high throughput drug exposure assay in 96-well plates. Bacterial survival was assessed by growth on LB plates. We identified 14 drug candidates (tosufloxacin, colistin, sparfloxacin, moxifloxacin and gatifloxacin, enrofloxacin and sarafloxacin, octodrine, clofoctol, dibekacin, cephalosporin C, pazufloxacin, streptomycin and neomycin), which had high anti-persister activity. Among them, tosufloxacin and colistin had the highest anti-persister activity and could completely eradicate *E. coli* persisters in 3 days *in vitro* while the current UTI antibiotics failed to do so. Our findings may have implications for the development of a more effective treatment for UTIs.

## 1. Introduction

Urinary tract infections (UTIs) are the second most common healthcare-associated infection [1] and account for about 25% of all common infections [2]. Uropathogenic *Escherichia coli* (UPEC) is the most dominant UTI pathogen, which is responsible for about 80%–90% of community-acquired UTIs and 30%–50% of nosocomially-acquired UTIs [3]. Within 6 months after an initial UTI, about one-fourth of women experience a second infection and many individuals have multiple, recurrent UTIs throughout their lives [4,5]. The bacteria associated with the recurrent UTI are often phenotypically or genotypically identical to the bacterial strain that caused the initial infection [6,7,8,9,10]. These findings suggest that the tendency of UTI to recur is due to relapse of quiescent UPEC persister bacteria after antibiotic treatment [10].

Current antibiotics commonly used to treat UTIs include beta-lactam antibiotics, sulfa drugs (trimethoprim-sulfamethoxazole), quinolones, aminoglycosides, nitrofurantoin, and fosfomycin [11]. However, these antibiotics are not very effective for treating UPEC persistent infections as the persister bacteria reside intracellularly and are tolerant to them [12]. To identify more effective drugs active against UPEC persisters for improved treatment of recurrent UTIs, in this study, we screened a clinical drug library [13] on stationary phase persisters of UPEC strain UTI89 and identified 14 drug candidates that had high anti-persister activity.

## 2. Results and Discussion

### 2.1. Identification of 14 Drug Candidates with High Anti-Persister Activity

**Figure 1:**
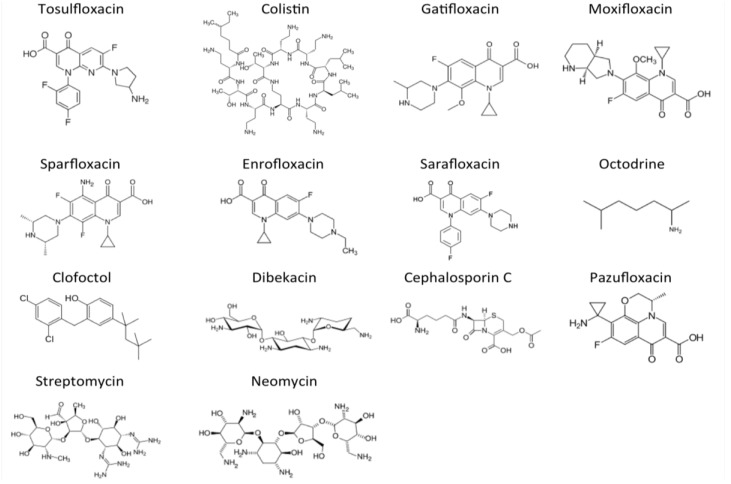
Structures of the 14 drug candidates with activity against *E. coli* UTI89 persisters.

We adopted a sequential drug exposure approach to identify drugs that had activity against UPEC persisters over time. After 3 day drug exposure, five compounds (tosufloxacin, colistin, sparfloxacin, moxifloxacin and gatifloxacin) were found to have high activity against UPEC persisters and produced no colonies on LB plates, and two compounds (enrofloxacin and sarafloxacin) had intermediate activity that produced small and faint growth on LB plates (Table 1). When the drug exposure was extended to 7 days, an additional seven drugs (octodrine, clofoctol, dibekacin, cephalosporin C, pazufloxacin, streptomycin and neomycin) were found to kill all UPEC persisters, with no growth detected on LB plates (Table 1). The MICs of the 14 drug candidates are shown in Table 1. The structures of the 14 drug candidates that showed anti-persister activity are shown in Figure 1. 

**Table 1:** Activity of 14 drug candidates from the clinical drug library that are active against stationary phase *E. coli* UTI89 persisters.

Drug name (50 μM)	MIC(μM)	Viability of Bacteria after 3 or 7 Days of Drug Exposure ^a^		CFU/mL of Bacteria after 3 or 5 Days of Drug Exposure ^b^	
		3 Days	7 Days	3 Days	5 Days
Tosulfloxacin	0.15	−	−	0	0
Colistin	0.6	−	−	0	0
Gatifloxacin	0.6	−	−	0	0
Moxifloxacin	0.6	−	−	0	0
Sparfloxacin	0.3	−	−	6000	0
Enrofloxacin	0.3	+/−	−	30,000	3300
Sarafloxacin	0.3	+/−	−	0	0
Octodrine	≥10	+	−	>50,000	0
Clofoctol	10	+	−	12,000	2300
Dibekacin	≥40	+	−	>50,000	0
Cephalosporin C	10	+	−	4000	0
Pazufloxacin	0.3	+	−	39,000	2700
Streptomycin	≥40	+	−	>50,000	1000
Neomycin	≥40	+	−	41,000	0
Drug-free control	−	+	+	1,500,000,000	760,000,000

^a^ Stationary phase *E. coli* UTI89 bacteria that survived ofloxacin treatment were treated with different drug candidates for 3 days or 7 days, and the viability of the bacteria was determined by transfer to LB plates using a 96-pin replicator or by CFU count on LB plates; ^b^ CFU data of the surviving bacteria after drug (50 μM) treatment with the 14 persister-active drug candidates. “−” No colonies grew on LB plates after drug exposure. “+/−”Small and faint colonies grew on LB plates after drug exposure. “+” Obvious colonies grew on LB plates after drug exposure.

### 2.2. Tosufloxacin and Colistin Showed the Highest Anti-Persister Activity

Based on the results of the primary screen, we selected these 14 drug candidates that showed anti-persister activity for rescreens, and the results were found to be reproducible. Next, CFU counts after 3 days and 5 days of drug exposure in Eppendorf tubes was also used to validate and rank the relative activity of the effective drug candidates identified using the 96-well plate method. We found that tosufloxacin, colistin, sarafloxacin, moxifloxacin and gatifloxacin eradicated all UPEC persisters after 3 day drug exposure, while cephalosporin C, neomycin, sparfloxacin, dibekacin, and octodrine killed all persisters after 5 day drug exposure. However, four drug candidates (clofoctol, pazufloxacin, streptomycin and enrofloxacin) only reduced the CFU of the UPEC persisters to a relative low level without complete eradication (Table 1).

### 2.3. Comparison of the Persister-Active Drug Candidates with Current UTI Antibiotics in Their Ability to Kill UPEC Persisters

**Figure 2:**
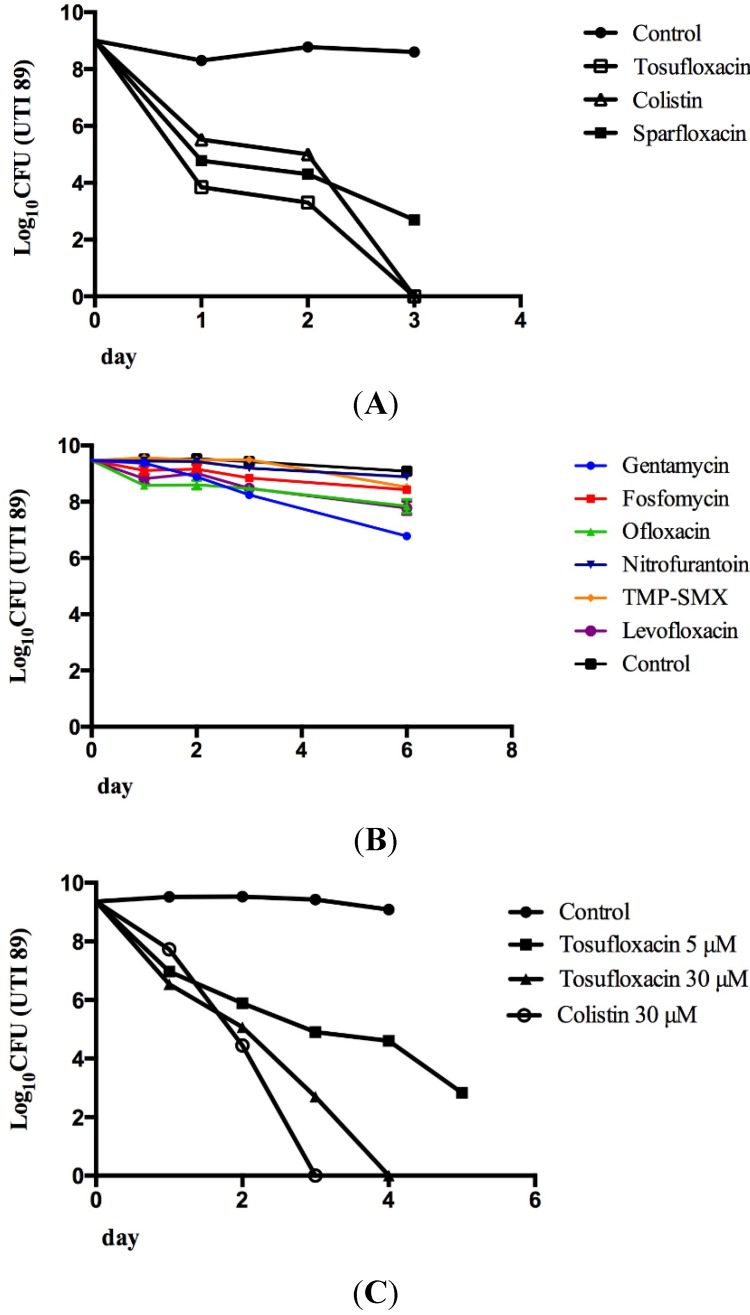
Activity of persister active drug candidates and selected conventional UTI antibiotics against *E. coli* UTI89 persisters. *E. coli* UTI89 stationary phase culture was treated with ofloxacin (5 μg/mL) for 4 hours to enrich persisters. Then, the surviving persisters from the treatment were subjected to drug exposure with different antibiotics as described in the text. The bacterial viability was determined by CFU count. (**A**) Tosulfloxacin (50 μM) and colistin (50 μM) killed persisters more effectively than sparfloxacin (50 μM) and completely eliminated persisters after 3 days; (**B**) Antibiotics commonly used to treat UTIs had poor activity against the UTI persisters. The final concentration of the UTI antibiotics including gentamicin, fosfomycin, ofloxacin, levofloxacin, nitrofurantoin and TMP-SMX, was all 50 μM. TMP-SMX is the combination of trimethoprim and sulfamethoxazole in a ratio of 5:1; (**C**) Activity of tosufloxacin (5 and 30 μM) and colistin (30 μM) at a lower concentrations against stationary phase *E. coli* UTI89 culture.

Among the five drugs that killed dormant persister cells over a 3-day period, four (tosufloxacin, sarafloxacin, moxifloxacin and gatifloxacin) are quinolone antibiotics and the other is colistin or polymyxin E. Thus, we compared tosufloxacin and colistin with antibiotics that are commonly used to treat UTIs including trimethoprim-sulfamethoxazole (TMP-SMX), nitrofurantoin, gentamicin, fosfomycin, sparfloxacin, ofloxacin, and levofloxacin, all at 50 μM, for their ability to eradicate UPEC persisters. Interestingly, tosufloxacin and colistin showed outstanding anti-persister activity, eradicating all persister bacteria of UTI89 stationary culture in 3 days (Figure 2A). Sparfloxacin had some activity against persisters, but its anti-persister activity was not as high as tosufloxacin or colistin (Figure 2A). Gentamicin, fosfomycin, ofloxacin, levofloxacin, nitrofurantoin and TMP-SMX (50 μM) commonly used to treat UTIs had no obvious activity against UPEC persisters after even 6 days of drug exposure (Figure 2B).

Tosufloxacin is a fluoroquinolone antibiotic that is characterized by having the 2,4-difluorophenyl and 3-amino-1-pyrrolidinyl groups in its structure. It has been reported that compounds with a 2,4-difluorophenyl group at the N-1 position in the quinolone nucleus exhibited good bactericidal activities [14]. Here we found that tosufloxacin showed the highest activity among the quinolone drugs against UPEC persisters, which may be due to the action of its unique side chain structure of the 2, 4-difluorophenyl group at the N-1 position. Future studies are needed to test this possibility. Colistin is a multicomponent antibiotic that is bactericidal to Gram-negative bacteria and acts by interfering with the structure and function of the outer and cytoplasmic membranes [15], and has a role in intravenous treatment of multidrug-resistant bacterial infections [16]. Interestingly, we found that colistin also had high anti-persister activity in this study. This finding is consistent with other observations that drugs that disrupt membrane, such as daptomycin and clofazimine, have good activity against persister bacteria [17].

UPEC persisters are the primary cause of UTI recurrence in humans [10], thus studies examining whether existing clinical drugs effectively eliminate UPEC persisters represent a rapid and efficient way to address this problem. Since clinical drugs have relatively clear safety and pharmacokinetic profiles, the effective drug candidates can be quickly applied in clinical setting. Here we identified tosufloxacin and colistin to have excellent anti-persister activity *in vitro* (Figure 2). Previous studies from Japan have demonstrated that tosufloxacin was highly effective for treating UTIs with efficacies of 100% and 73% for acute and chronic UTIs, respectively [18,19]. However, tosufloxacin was not known to have anti-persister activity before this study. Tosufloxacin was approved in 1990 in Japan, but has been associated with thrombocytopenia, nephritis and hepatoxicity and has not gained FDA approval in the US. The extraordinary activity of tosufloxacin against UPEC persisers might explain its high clinical efficacy, especially for the chronic UTI. Further studies are needed to evaluate tosufloxacin and colistin in animal models of UTI to determine the optimal regimens that better eliminate UPEC persisters than the current UTI antibiotics before clinical studies.

## 3. Experimental Section

### 3.1. Bacterial Strain

UTI89 is an uropathogenic *E. coli* strain isolated from a woman with cystitis (acute bladder infection) [20]. We chose this strain in this study as a representative clinical strain since it has also been demonstrated to cause cystitis and persistence despite antibiotic treatment in a murine urinary tract infection model [21]. The UTI89 strain was cultured in LB broth overnight to stationary phase with shaking at 37 °C. Then the overnight culture (~3 × 10^9^ CFU/mL cells) was treated with ofloxacin (5 μg/mL) for 4 hours to kill growing bacteria and enrich persisters.

### 3.2. Antibiotics and the Clinical Drug Library

Tosufloxacin, colistin, sparfloxacin, gentamicin, fosfomycin, ofloxacin, levofloxacin, nitrofurantoin and trimethoprim-sulfamethoxazole (TMP-SMX) were purchased from Sigma and dissolved in appropriate solvents to form stock solutions. All antibiotic stocks were filter-sterilized using a 0.2 mm filter.

The clinical drug library consisting of 1524 pharmacologically active compounds, which were either FDA-approved drugs or drugs approved for use abroad, was prepared as 10 mM stock solutions and 1 mM working stock in dimethyl sulfoxide (DMSO) [13], leaving the first and last columns in each plate for controls.

### 3.3. Screening of the Clinical Drug Library in the UTI89 Stationary-Phase Persister Model

The persister bacteria that survived the ofloxacin treatment (prepared as above) were washed twice and resuspended in 3-(*N*-morpholino) propanesulfonic acid (MOPS) buffer and transferred to 96-well plates for drug screens. To qualitatively determine the effect of FDA-approved drugs on UTI persisters, each compound from the working stock (5 μL) was added to the persister bacteria in the screening plate. Each well was adjusted to 100 μL with MOPS buffer to achieve a final drug concentration of 50 μM. The plates were incubated at 37 °C without shaking. At 3 or 7 days of drug exposure, a 96-pin replicator was used to transfer the bacterial suspension onto LB agar plates, to monitor the bacterial survival after the drug exposure.

### 3.4. Validation of Active Drug Candidates by Colony Forming Unit (CFU) Count

The surviving persisters from the ofloxacin treatment were dispensed into 1.5 mL Eppendorf tubes in 1 mL followed by addition of drugs at the final concentration of 50 μM. At different time points of drug exposure, 100 μL bacterial suspension was removed from the tubes followed by washing in PBS. Serial dilutions were performed and 10 μL of each dilution was spotted onto LB agar plates to get CFU count after incubation overnight at 37 °C.

## 4. Conclusions

In this study, we screened a clinical drug library and identified 14 drug candidates that had high anti-persister activity. Tosufloxacin and colistin were found to have the highest anti-persister activity and could completely eradicate *E. coli* persisters while the current UTI antibiotics could not. These newly identified anti-persister drugs may have implications for effective treatment of UTIs and further evaluation in the animal models of persistence is warranted.
